# Modeling Growth Dynamics of *Lemna minor*: Process Optimization Considering the Influence of Plant Density and Light Intensity

**DOI:** 10.3390/plants14111722

**Published:** 2025-06-05

**Authors:** Jannis von Salzen, Finn Petersen, Andreas Ulbrich, Stefan Streif

**Affiliations:** 1 Faculty of Agricultural Sciences and Landscape Architecture, University of Applied Sciences Osnabrück, Am Krümpel 31, 49090 Osnabrück, Germany; finn.petersen@hs-osnabrueck.de (F.P.); a.ulbrich@hs-osnabrueck.de (A.U.); 2 Automatic Control and System Dynamics Laboratory, Institute of Automation, Chemnitz University of Technology, 09126 Chemnitz, Germany; stefan.streif@etit.tu-chemnitz.de; 3 Department of Bioresources, Fraunhofer Institute for Molecular Biology and Applied Ecology, 35392 Giessen, Germany

**Keywords:** *Lemna*, duckweed protein, growth modeling, process optimization, light intensity, vertical farming, artificial lighting, sustainable agriculture, light use efficiency, indoor farming

## Abstract

The production of duckweed (Lemnaceae) as a novel protein source could make a valuable contribution to human nutrition. The greatly reduced habitus of duckweed enables simple cultivation with extremely low space requirements, making this free-floating freshwater plant ideal for substrate-free and vertical cultivation in controlled environment agriculture. Of particular importance in the design of a plant-producing Indoor Vertical Farming process is the determination of light intensity, as artificial lighting is generally the most energy-intensive feature of daylight-independent cultivation systems. In order to make the production process both cost-effective and low emission in the future, it is, therefore, crucial to understand and mathematically describe the primary metabolism, in particular the light utilization efficiency. To achieve this, a growth model was developed that mathematically describes the combined effects of plant density and light intensity on the growth rate of *Lemna minor* L. and physiologically explains the intraspecific competition of plants for light through mutual shading. Furthermore, the growth model can be utilized to derive environmental and process parameters, including optimum harvest quantities and efficiency-optimized light intensities to improve the production process.

## 1. Introduction

The global human population is expected to grow to 9.6 billion people by 2050 [1]. As a result, the demand for protein will increase dramatically [2,3]. Due to the climate-damaging consequences and the high land requirements associated with the production of animal protein, the production of high-quality plant protein represents an important factor in the development of current and future food production. At the same time, a higher share of the global population lives in cities [4] which leads to an increasing demand for locally and regionally produced high-quality food in urban regions.

An innovative way to counteract these problems could be the cultivation of duckweed in controlled environment agriculture (CEA) systems. Duckweed is the fastest-growing flowering plant in the world [5] and, depending on the species and cultivation can reach protein contents of 40% in dry weight [6,7,8]. The amino acid profile of duckweed protein is ideal for human consumption [9]. In addition, no significant health disadvantage or unpleasant flavor was found for the consumption of the species *Lemna minor* L. in comparison with spinach [10]. Duckweed is gaining importance as an aquatic crop, as evidenced by the authorization of specific species as foodstuffs in the European Union [11]. The greatly reduced habitus and the free-floating aquatic nature of the plants enable easy cultivation with low space requirements without the need for substrates, which have a significant impact on the ecological footprint of other horticultural crops [12]. This makes duckweed ideal for cultivation in Indoor Vertical Farming (IVF) concepts.

In IVF concepts, the energy consumption due to artificial lighting is significantly higher compared to open-field or greenhouse cultivation, making it crucial to minimize energy use in the cultivation process. To economically operate CEA systems for duckweed production, it is necessary to optimize the cultivation process by optimizing all relevant abiotic and biotic growth parameters. This involves minimizing resource and energy inputs while maximizing biomass yield and quality. Studies have explored various approaches to designing such a duckweed cultivation system and process [13,14,15].

It is essential to understand the impact of biotic and abiotic factors on biomass yield and quality to achieve these goals. This understanding can be facilitated by employing growth models to describe these interactions. As in all biological systems, duckweed populations are subject to ecological limits on growth, arising from intraspecific competition for resources. As competition intensifies through the growth of biomass, the maximum growth rate physiologically achievable is constrained. Consequently, plant density emerges as a crucial biotic factor influencing the growth rate. By accurately describing this relationship, it becomes feasible to optimize plant density, which in turn enables the optimization of the harvesting process [14].

In the context of the design of production processes for phototrophic organisms, such as plants, the determination of light intensity is of particular importance, given its significant influence on the growth rate. This is also applicable to different species of duckweed [16,17,18]. Furthermore, the highest energy demand in daylight-independent cultivation systems is typically due to artificial lighting [12]. Precise knowledge of the effect of light intensity on plant growth dynamics is therefore crucial.

A range of growth models for duckweed have been developed, differing in their mathematical relationship between plant density and growth rate, as well as in their depictions of the effects of abiotic factors [14,19,20,21,22,23,24]. Van Dyck et al. [23] developed a model to describe the effect of light intensity on the potential growth rate of *L. minor*. This model was developed using growth data from batch cultures. In contrast, this study investigates growth in a recirculating culture system where sufficient water movement ensures a homogeneous distribution of plants and nutrient solutions across the culture. As a result, the growth-inhibiting effect of high plant density is considered to be primarily a result of mutual shading, which is less effective at higher light intensities due to deeper penetration of light into the plant layer. Consequently, it can be hypothesized that there is a strong interaction effect between light intensity and plant density on growth dynamics resulting from the perception that the average light intensity at the individual plant is composed of the light intensity at the water surface and the layer thickness. This could indicate that a newly developed model may be able to describe the processes in the recirculating culture system better than the model of Van Dyck et al. [23]. Although the composed effect of light intensity and plant density could also affect morphology, the focus of this study is on growth dynamics and population ecology, as the growth rate is crucial for the production of protein-rich biomass.

Furthermore, process-relevant parameters are derived from the developed model, such as the optimum plant density or indicators for the efficiency of biomass production. In addition, product quality in the form of crude protein content was investigated at different light intensities and plant densities. The results obtained from this study have the potential to facilitate the planning and operation of duckweed cultures, thereby enhancing cost and energy efficiency while maintaining consistent product quality in the future based on scientific findings.

## 2. Methods

### 2.1. Plant Material

The duckweed species *L. minor*, clone 9441 (RDSC Database of Duckweed Clones [25]), originally isolated in Germany, was used for the experiment. The plants in this study originate from the stock collection of the University of Jena, Germany. Prior to the experiment, the plants were pre-cultured for seven days under the environmental conditions in which they would be grown in the experiment, including the corresponding light intensities. The plants had already been cultivated in the experimental culture system prior to the preculture. So most of the environmental parameters were not changed by the start of the preculture. It was determined that a preculture duration of seven days was sufficient, based on previous experience from other experiments [16,26].

### 2.2. Re-Circulating Controlled Agriculture Environment System

**Figure 1:**
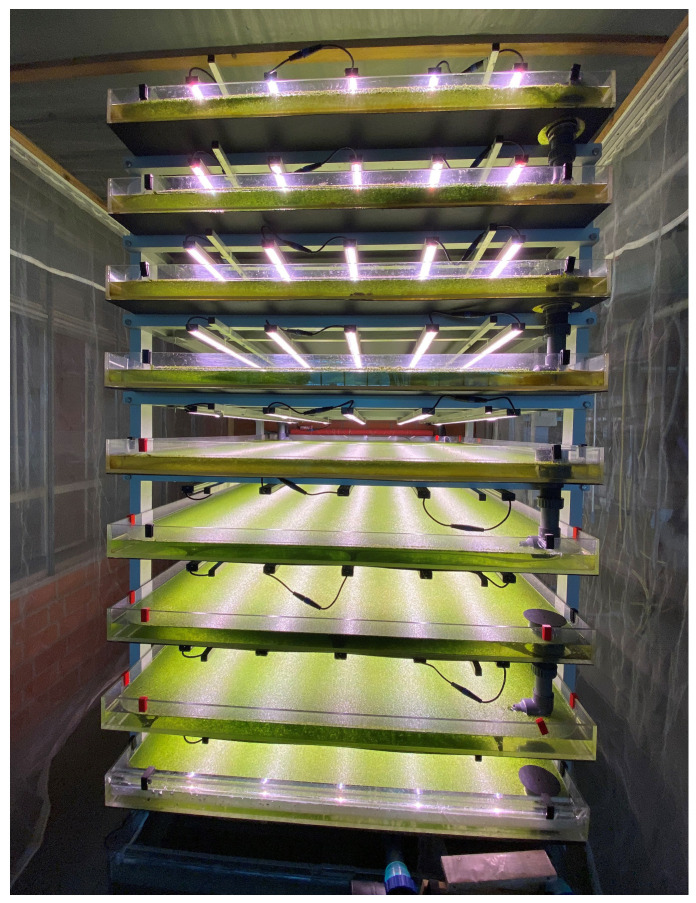
Fully planted indoor vertical farm for duckweed production in operation [13].

The study was conducted at the University of Applied Sciences Osnabrück, Germany, in a vertical, recirculating culture system, as described in detail by Petersen et al. [13]. The system is comprised of nine vertically stacked production basins and a nutrient solution reservoir that is placed on the floor. Each production basin is equipped with five LED modules (FlexPro, Spectrum S4—daylight-like, SANlight GmbH, Schruns, Austria), allowing for independent adjustment of the light intensity for each production basin (Figure 1).

Nutrients were dosed automatically based on the electrical conductivity (EC; accuracy: 100 µS·cm^−1^) using the Pro Controller and PeriPods M4 (Bluelab Corporation Ltd., Tauranga, New Zealand).

**Figure 2:**
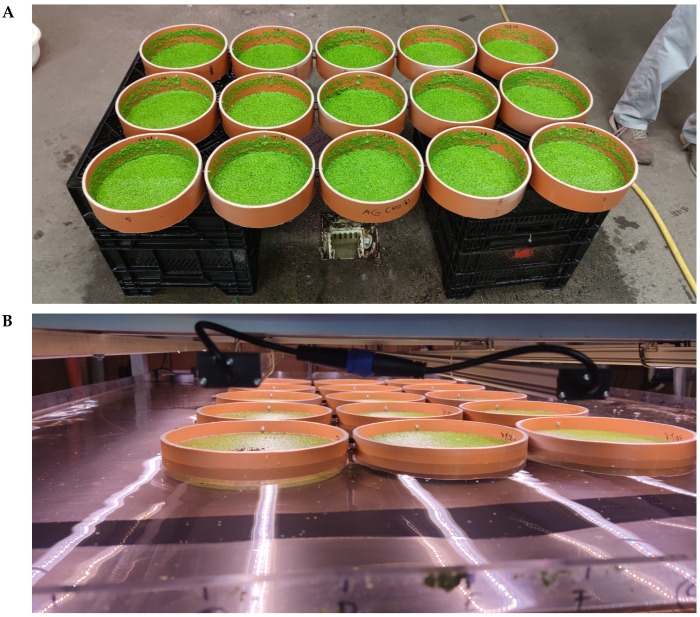
Ring battery, consisting of 15 plastic rings bolted together, which divides a production basin into 15 evenly illuminated areas of the same size of 0.0443 m^2^ (**A**) and position on the production basin (**B**).

To maximize the number of variants and repetitions, the duckweed was cultivated in plastic rings within the basins. The lower eight production basins of the production system were equipped with 15 plastic rings each. This resulted in a total of 120 rings. Each plastic ring had an inner diameter of 237.6 mm, resulting in a cultivation area of 0.0443 m^2^ per plastic ring subarea. To facilitate the stocking and harvesting, the rings were equipped with a nylon mesh (mesh size < 1 mm) on the underside and were bolted together at each tier, resulting in 8 ring batteries as shown in Figure 2.

The ring batteries were positioned at the center of the production basins on approximately 1.5 cm high pillars beneath the middle three LED elements to ensure a near-uniform light distribution and nutrient solution exchange. To minimize edge effects caused by plants adhering to the plastic ring walls, the following procedure was adopted. After the placement of the ring batteries on the basins, the inner plastic ring walls were rinsed with a laboratory spray bottle containing nutrient solution. Subsequently, the water level of the basins was raised slightly. The plants adhering to the walls of the plastic rings seen in Figure 2A were caused by lowering the water level of the basins to remove the ring batteries at the end of the experiment. The abiotic environmental factors employed in the experiment are presented in Table 1.

As a nutrient solution, a N-medium with a NO_3_^−^-N:NH_4_^+^-N ratio of 3:1 was used, as described in Petersen et al. [26] (Table 2).

### 2.3. Variants and Repetitions

The light intensity variants were configured to utilize a broad spectrum of different light intensities possible, given the power limitations of the LED modules and the culture system used. The density variants were selected to ensure that at high plant densities, a significant decrease in growth rate could be anticipated, thereby allowing for an estimation of the respiration rate. For estimating the magnitude of the plant density, the findings of Monette et al. [20] and Driever et al. [19] were utilized.

Six initial biomass density variants (D_0_) were selected (10, 42, 74, 106, 138, and 170 gDW·m^−2^), along with four light intensity variants (42.7, 88.2, 123.9, and 166.2 µmol·s^−1^·m^−2^). This results in a total of 24 different combinations, each replicated five times, leading to 120 growth trials (Table 3).

The light intensity variants were randomized across the eight production basins of the culture system leading to two basins per light intensity variant. Within each pair of ring batteries per light intensity variant, the 30 plastic ring subareas containing the six plant density variants in five repetitions each, were randomly distributed. This compensated for any micro gradients in the environmental conditions in the cultivation system.

The methodological approach for calculating the average light intensities over the specific plastic ring subareas entailed the utilization of spatial interpolation and statistical analysis. In the initial stage of the process, the spatial distribution of the light intensity data was modeled using an anisotropic spherical variogram, with the objective of capturing the spatial dependencies across the various measurement points. Light intensity was measured at 65 points for each basin. Kriging interpolation was conducted on a 100 × 100 grid, resulting in the generation of a continuous light-intensity field. To perform the averaging of partial surfaces, virtual circles with predefined diameters of 237.6 mm, corresponding to the inner diameters of the plastic rings, and predefined center points, corresponding to the center points of the plastic rings were positioned over the interpolated surface. The mean intensity within each circle was calculated by selecting all grid points falling inside the circle. The standard deviation of the light intensity values within these circles was also calculated. This method ensures spatial interpolation precision and facilitates detailed local intensity analysis across the specified grid. The calculations were conducted using R version 4.3.3 and RStudio version 2024.12.0+467 [30,31,32,33,34,35,36,37,38]. The script is attached in the Supplementary Files.

The interpolated areas of the eight basins, in addition to the mean light intensities and the standard deviations within the ring sub-areas, are attached in the Supplementary Files.

### 2.4. Data Collection

At the beginning of the experiment, the fresh weight (FW) of the duckweed was measured. Additionally, at the end of the experiment, the fresh and dry weight (DW) as well as the crude protein content were recorded. For statistical data analysis, the dry weight instead of the fresh weight is used, as it exhibits less variability. The dry weight content is calculated according to Equation (1).

(1) $$\%\mathrm{DW}=\frac{\mathrm{DW}}{\mathrm{FW}}$$

To determine the fresh weight (FW) at the start and the end of the experiment, the duckweed fronds were washed with cistern water (rainwater) to remove any adhering algae and nutrient solution and subsequently moderately centrifuged (rotation speed: 1440 rpm, radius = 105 mm, oneConcept Top Spin Compact, Chal-Tec GmbH, Berlin, Germany) for a duration of 5 min, with a centrifugal force of 100–240 g, depending on the distance of the centrifuged duckweed to the centrifuge edge. Sedimentation of cellular contents or even damage to the cells is considered to be very unlikely at centrifugation of this intensity [39].

Dry weight (DW) was determined via oven drying (TUH 75/100, Heraeus Holding GmbH, Hanau, Germany) at 60 °C for 72 h according to DIN EN ISO 20079:2006-12 [40].

The dry weight content is determined destructively. Consequently, the plant dry weight and plant density cannot be measured exactly at the start of the trial. For this purpose, a long-term dry weight content of 6%, which was achieved in this IVF for *L. minor* [13], was assumed and the required fresh masses were weighed on this basis. In addition, five samples of exactly 20 g fresh weight each were taken from each preculture for each light intensity variant at the start of the trial, centrifuged, and dried. The dry weight and plant density at the beginning of the experiment were then corrected using the mean dry weight content of these samples of the corresponding light-intensity variant. This method entails a slight variation in the initial plant densities of the light variants.

### 2.5. Nutrient Solution

To analyze the nutrient content and composition of the nutrient solution, samples were taken daily throughout the experiment. To generate representative nutrient solution samples for the entire IVF, composite samples were taken containing approximately 100 mL from each production basin and 200 mL from the reservoir. Approximately 200 mL of these composite samples were filtered, and the remainder was preserved as an unfiltered retention sample, frozen at −18 °C. The nitrate and ammonium content were determined photometrically [27,28] using a Lambda 25 (PerkinElmer, Waltham, MA, USA). The concentrations of phosphorus, potassium, sulfur, magnesium, calcium, sodium, manganese, zinc, iron, boron, and copper in the nutrient solution samples were determined by ICP-OES [29] using an ICAP 7400 (Thermo Fisher Scientific, Waltham, MA, USA).

The temperature, and pH of the nutrient solution were logged hourly by sensors connected to a Pro Controller (Bluelab Corporation Ltd., Tauranga, New Zealand). The electrical conductivity was measured daily using an EC meter. The daily unfiltered nutrient solution sample was used for this measurement.

### 2.6. Determination of the Crude Protein Content

The nitrogen content of the dried samples was determined by applying the Dumas combustion method [41,42] using an elemental analyzer (FP628, Leco, Saint Joseph, MI, USA). Crude protein content was calculated using the factor 6.25 [43,44].

### 2.7. Growth Model Development, Fitting and Comparison

A mathematical model was developed to describe the growth of the duckweed dry weight per area. The model describes the physical and physiological processes occurring within the plant cover and is intended to serve as a basis for optimizing the cultivation process, particularly by determining the optimal light intensity and the dependence of the harvest interval on this factor.

The dynamics of the plant density as dry weight per area (D) during the experiment are modeled as an ordinary differential equation depending on a function of the light intensity at the plant layer surface, which is equivalent to the water surface (I_s_) and plant density (D) over the seven-day trial period:

(2) $$\frac{\mathrm{dD}}{\mathrm{dt}}=f\left(I_{s},D\right).$$

The growth of duckweed is limited by intraspecific competition for resources. The limit density (D_L_), which represents the population-ecological capacity limit of the cultivation system, is also determined. D_L_ is obtained as the limit of the solution to Equation (2) as the variable t approaches infinity:

(3) $$D_{L}=\underset{t\to \infty }{\lim}D\left(t\right)$$

The dependence of the growth rate on other environmental factors is not considered in this approach. The water temperature, pH and nutrient concentrations showed only a minor deviation throughout the experimental phase (Table 1), due to the recirculating operation of the culture system with a sufficient circulation rate of 0.2–0.3 h^−1^.

Furthermore, to simplify the development of the growth model, it was assumed that the availability of CO_2_ was uniform in the area distribution and did not depend on the thickness of the plant layer.

The influence of the spectral light composition, both in the area distribution and in relation to the depth in the plant layer, was not taken into account for simplification, as Petersen et al. [16] were unable to determine any influence of the spectral composition on both the growth rate and the crude protein content of *L. minor*.

The model fitting was achieved by minimizing the residual sum of squares for D_7_. The optimization was conducted using Rversion 4.3.3 and RStudio version 2024.12.0+467 [30,31,45,46,47,48]. The script is attached in the Supplementary Files.

Many other growth models that address the effects of light intensity and plant density or plant stock architecture, particularly in terrestrial plants, incorporate the Leaf Area Index (LAI). This procedure is unusual for duckweed, as the plant density in dry weight per area is easier to quantify in duckweed cultures.

However, when the leaf mass per area (LMA) is taken into consideration, the leaf area index (LAI) could in principle be estimated from the plant density as follows:

(4) $$\mathrm{LAI}=\frac{D}{\mathrm{LMA}}$$

The LMA for *L. minor* was not analyzed in the present study. To estimate the value to a sufficient extent, 11.5 g·m^−2^ can be assumed, which was determined for *L. minor* RDSC5512 [49,50]. However, the extent to which the LMA is constant or is also influenced by environmental factors is questionable. For This reason, D was modeled instead.

The biomass growth over the test period of seven days was simulated with a step size of 0.05 days using the Dormand-Prince method.

In addition to the developed model, the growth data from the cultivation experiment were fitted to the model of Van Dyck et al. [23] with the parameters D (labeled B in source), r_phot,i_ (labeled r in source), r_resp_ (homologous to r_d_ in source), E (labeled τ in source) and I (labeled L in source) (Equation (5)) to determine whether the consideration of the physical and physiological processes provides a more accurate description of the growth kinetics than this previous approach.

(5) $$\frac{\mathrm{dD}}{\mathrm{dt}}=D\cdot \left(r_{\mathrm{phot},i}\cdot \left(1-\frac{D}{h_{D}}\right)\cdot E\cdot \left(\frac{I}{K_{I}+I}\right)-r_{\mathrm{resp}}\right)$$

The model fitting was conducted using R version 4.3.3 and RStudio version 2024.12.0+467 [30,31,45,46,47,48]. The script is attached in the Supplementary Files.

### 2.8. Methods of the Model Validation

To validate the model and test its applicability to longer cultivation phases, a long-term cultivation trial was carried out in addition to the growth trial already described. This trial was conducted in the same IVF environment and under the same environmental conditions at light intensities of 58.4, 109.2, 136.0 and 168.1 µmol·m^−2^·s^−1^. Each light intensity variant was replicated four times, with the same initial plant density of approximately 17 gDW·m^−2^ (10 gFW per plastic ring subarea). The initial biomass differed marginally due to the initial estimation and subsequent correction of the dry matter content. In an interval of two weeks, one test culture per light intensity variant was centrifugated and dried as described in the methodology to determine the dry matter content. This results in a total experiment duration of eight weeks. The remaining cultures were washed, centrifuged, and weighed as described before and returned to the experiment. To keep both the plants and the plastic rings clean and free of debris over the long term, they were also washed and centrifuged in the weeks between the weighing schedules. The plant density was determined from the weighed fresh masses by applying the dry matter content of the harvested trial. The growth data collected in this manner was compared with the growth model that was developed and the growth model from Van Dyck et al. [23]. Apart from light intensity, the values of the model parameters were copied from the model fitting, on the basis that the environmental factors were identical to those in the main experiment.

R^2^ was employed to assess the model’s accuracy in fitting the validation data by comparing the variance of the observed plant densities to the variance of the discrepancies between these observed values and those predicted by the model.

The simulation of the growth models and the R^2^-calculation were conducted using R version 4.3.3 and RStudio version 2024.12.0+467 [30,31,36,46,51]. The scripts are attached in the Supplementary Files.

### 2.9. Methods of the Model Utilization for Process Optimization

The adapted growth model was employed to determine additional parameters that are crucial for planning and controlling the cultivation process. A semi-continuous production process was assumed, in which a portion of the crop is removed at a constant harvest interval (Δt_H_). The remaining duckweed biomass remains in the production area to grow again until the next harvest interval.

A key assumption of the process optimization is that Δt_H_ is primarily dependent on the procedural implementation possibilities of the harvesting process. Depending on the degree of automation, the harvesting process can be subject to working times and personnel deployment. It can be assumed that a maximum yield can be achieved with continuous harvesting, although this may be technically difficult to implement, especially as there is currently no precise, automated, non-invasive in-time measurement method for determining the plant density (D) that can also measure reliably at higher plant densities. There have been approaches for this measurement, which are based on camera data [52,53]. However, it should be noted that the accuracy of these measurements is likely to be compromised as soon as the fronds overlap, which occurs at plant densities that exceed 16 gDW·m^−2^ [53]. Δt_H_ can also vary due to operational processes, such as maintenance or cleaning of components of the cultivation or harvesting system, or the availability of workforce on vacation or weekend days. For this reason, the calculations were carried out with a variety of different harvesting intervals.

The additional parameters for the process management are the yield-optimized plant density that remains in the cultivation system after a harvesting process (D_opt_) and the average daily harvest yield at a density-optimized cultivation process (${\dot{m}}_{\max}$). It can be postulated that D_opt_ is contingent upon both light intensity and harvest frequency. ṁ_max_ is assumed to be dependent on Δt_H_ and I_S_. An additional parameter is the light use efficiency (LUE). It indicates the conversion factor of photosynthetically active radiation -photons to dry biomass and is also assumed to be dependent on Δt_H_ and I_S_. Furthermore, the light intensity with the maximum LUE (I_LUEmax_) is determined. I_LUEmax_ is also assumed to be dependent on Δt_H_.

## 3. Results and Discussion

### 3.1. Growth Data

**Figure 3:**
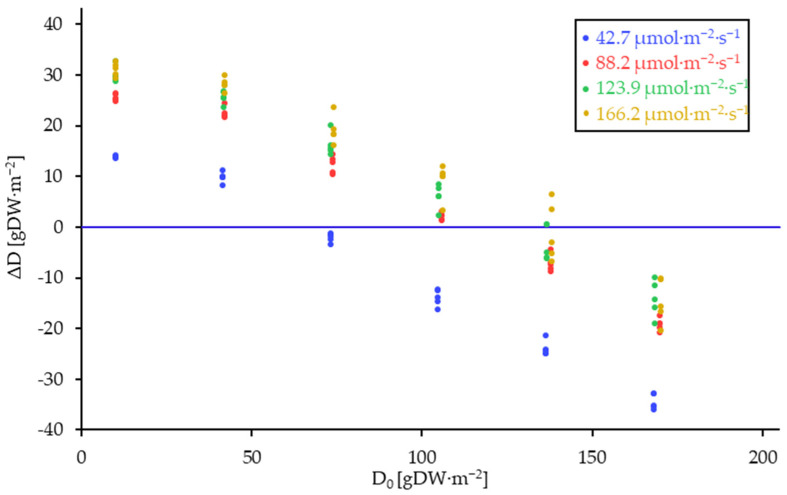
Plant density differences of *L. minor* (ΔD) over the trial duration of seven days. Grouped by light intensity, displayed in dependency of the initial plant density (D_0_). The data demonstrate a decline in biomass growth with increasing initial plant density. Furthermore, the growth data indicate a heightened biomass increase with elevated light intensity, with the 42.7 µmol·m^−2^·s^−1^ variant being particularly distinct from variants subjected to higher light intensities.

The difference in plant density over the trial period of seven days (ΔD) was calculated as the difference between the densities at the beginning and the end of the trial. The data was grouped according to light intensity and plotted in Figure 3 as a dependency of the initial plant density D_0_.

### 3.2. Growth Model

The variables and parameters used in the model development are summarized in Table 4.

The difference in plant density is simplified and regarded exclusively as the sum of plant physiological processes involved in the primary metabolism. In duckweed, photorespiration is of minor importance [54], so the primary metabolism can be described as the sum of photosynthesis and respiration. It is assumed that the photosynthesis rate is dependent on the light intensity and influenced by the plant density due to intraspecific competition by mutual shading. Therefore, the photosynthesis rate is defined as the product of the intrinsic photosynthesis rate as relative plant density increases over time (r_phot,i_) and a reduction term (fphot(I_s_,D)), which describes the dependence of the photosynthesis rate on the light intensity at the water surface (I_s_) and the plant density (D). In the isothermal experimental setup presented here, the respiration rate defined as the relative plant density decrease over time (r_resp_) is considered constant as it is not affected by the light intensity or the plant density. This results in the growth model shown in Equation (6). Its general structure aligns with the models developed by Driever et al. [19], Van Dyck et al. [23] and Schmitt et al. [22].

(6) dDdt=D·rphot,i·fphotIs,D−rresp

According to Van Dyck et al. [23], the photoperiod can be described as a factor of fphot. Equation (6) can be expanded to include the photoperiod (E) as the proportion of illuminated time over the photoperiodic cycle (Equations (7) and (8)).

(7) $$E=\frac{t_{\mathrm{light}}}{t_{\mathrm{photoperiodic}\;\mathrm{cycle}}}$$

(8) dDdt=D·E·rphot,i·fphotIs,D−rresp

The light intensity decreases as the light travels a distance ($\tilde{h}$) through the plant layer. The attenuation of the light intensity when traveling a distance in plant matter can be described using Lambert Beer’s law as found in standard textbooks [55] (Equation (9)). The attenuation constant ε is defined as the proportion by which the radiation intensity decreases when a unit $\tilde{h}$ is transmitted by the radiation.

(9) $$I\left(\tilde{h}\right)=I_{S}\cdot e^{-\varepsilon \cdot \tilde{h}}$$

As a result, the photosynthesis rate of the plants also decreases by the depth within the plant cover.

The limitation of the photosynthesis rate as a function of the light intensity can be modeled as a Monod-type function according to Pasos Panqueva et al. [56] (Equation (10)). The half-saturation constant, denoted by k, is a measure of the light intensity at which the photosynthesis rate is reduced to half its value when the light is fully saturated.

(10) $$f_{\mathrm{phot}}\left(I\right)=\frac{I}{k+I}$$

To calculate the reduction in the photosynthesis rate as a function of the depth within the plant cover ($\tilde{h}$), Equation (9) can be inserted into Equation (10), leading to:

(11) $$f_{\mathrm{phot}}\left(I_{S},\tilde{h}\right)=\frac{I_{S}\cdot e^{-\varepsilon \cdot \tilde{h}}}{k+\left(I_{S}\cdot e^{-\varepsilon \cdot \tilde{h}}\right)}.$$

To calculate the average limitation of the photosynthesis rate of the entire plant layer instead of the limitation of the photosynthesis rate at a specific depth of the plant layer, Equation (11) is integrated from 0 to the thickness of the plant layer h, and then divided by h, which yields the following expression:

(12) fphotIS,h=∫0hIs·e−ε·h~k+Is·e−ε·h~dh~h=−ln⁡k+IS·e−ε·h+ln⁡k+ISε·h.

The thickness of the plant layer h is assumed to be proportional to the plant density D (Equation (13)). The proportionality is described by the proportionality constant c (Equation (14)). Particularly at low plant densities, the biomass is not distributed evenly over the area, but in the form of individual plants. This simplification is likely to become less significant as plant density increases.

(13) h∼D

(14) h=c·D

Substituting the definition for h from Equation (14) into Equation (12) results in:

(15) fphotIS,D=−ln⁡k+IS·e−ε·c·D+ln⁡k+ISε·c·D.

The product ε·c can be replaced by the new constant $\hat{\varepsilon }$ and yields the more compact equation:

(16) fphotIS,D=−ln⁡k+IS·e−ε^·D+ln⁡k+ISε^·D.

This results in the following complete growth model:

(17) $$\frac{\mathrm{dD}}{\mathrm{dt}}=D\cdot \left({E\cdot r}_{\mathrm{phot},i}\cdot \frac{-\ln\left(k+I_{S}\cdot e^{-\hat{\varepsilon }\cdot D}\right)+\ln\left(k+I_{S}\right)}{\hat{\varepsilon }\cdot D}-r_{\mathrm{resp}}\right)$$

The limit density of the duckweed culture (D_L_) is the saturation limit of the growth curve and is obtained as described in Equation (3).

### 3.3. Model Fitting

The plant densities at the beginning of the trial (D_0_), the plant densities at the end of the trial (D_7_), and the calculated mean light intensities at the culture surface per plastic ring (I_S_) were employed to estimate the model parameters r_phot,i_, r_resp_, $\hat{\varepsilon }$ and k. The estimated values of the parameters and the coefficient of the determination of the model fit are presented in Table 5.

**Figure 4:**
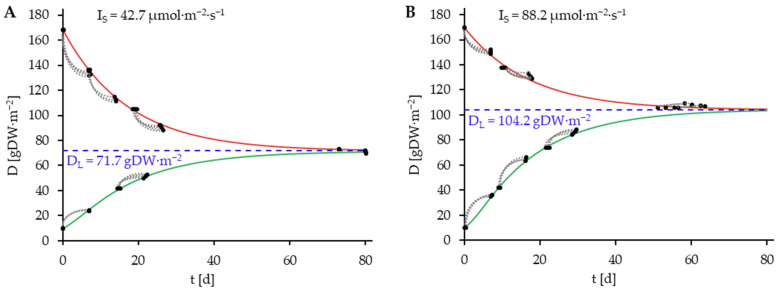

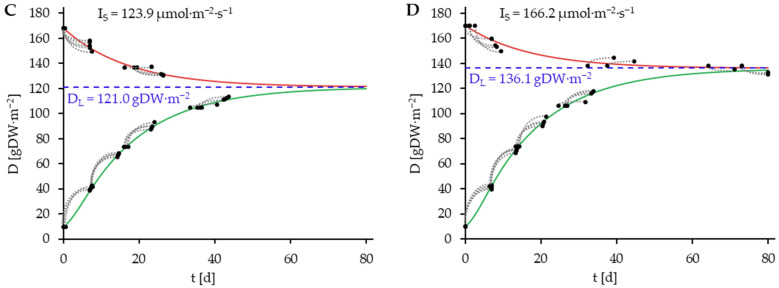
Growth data grouped by light intensity variant ((**A**): 42.7 µmol·m^−2^·s^−1^, (**B**): 88.2 µmol·m^−2^·s^−1^, (**C**): 123.9 µmol·m^−2^·s^−1^, (**D**): 166.2 µmol·m^−2^·s^−1^) and fitted growth model (Equation (17), Table 5), represented as growth curves, starting with the highest and lowest plant density variants. The simulation is conducted over a period of 80 days. Green lines indicate simulated positive plant growth at plant densities below the D_L_, red lines indicate simulated negative plant growth at plant densities above D_L_. Value pairs (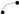) consisting of D_0_ and D_7_. Blue dashed lines represent D_L_.

**Figure 5:**
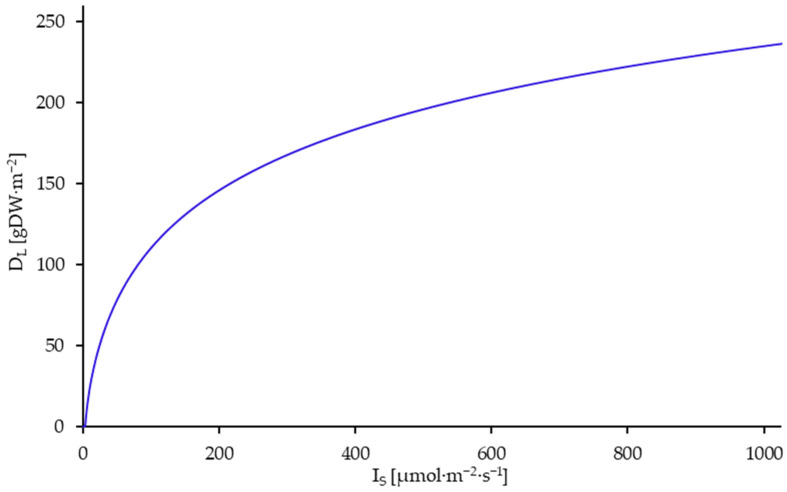
Limit density (D_L_) of *L. minor* dependent on the light intensity at surface level. Simulated from 0 to 1000 µmol·m^−2^·s^−1^ through the simulation of time trajectories until the saturation limit D_L_.

The growth curves for the light intensity variants (**A**: 42.7 µmol·m^−2^·s^−1^, **B**: 88.2 µmol·m^−2^·s^−1^, **C**: 123.9 µmol·m^−2^·s^−1^, **D**: 166.2 µmol·m^−2^·s^−1^) of the experimental data were generated based on the data from the fitted growth model (Equation (17), Table 5) and displayed in Figure 4. Additionally, the D_0_-D_7_ value pairs of the experimental data are also plotted on the graphs along with the D_L_-values (Equation (3), Figure 5). The determination of D_L_ was achieved through the simulation of a time trajectory until the saturation of D_L_.

It is apparent that the developed model, characterized by an R^2^ of 0.9950, provides a reliable explanation for the measured growth data. The assumptions and simplifications made during model development, such as the uniform availability of CO_2_ and nutrients within the plant layer or the description of the light saturation of photosynthesis as a Monod-type function, do not appear to have a profound negative effect on the reliability of the model.

It is apparent from the data that the population-ecological capacity limit of the cultivation system (D_L_) is also dependent on light intensity. This dependence was simulated based on the fitted growth model, as illustrated in Figure 5.

### 3.4. Results of the Model Validation

In addition to the fitting of the growth model developed in this work, the growth model described in Equation (5) by Van Dyck et al. [23] was fitted to the data. The estimated values of the parameters and the coefficient of the determination of the model fits are presented in Table 6.

**Figure 6:**
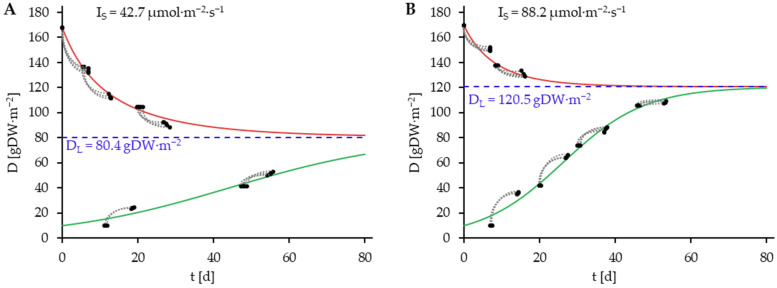

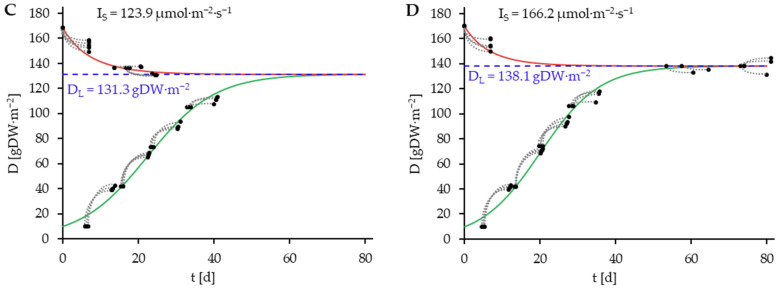
Growth data grouped by light intensity variant ((**A**): 42.7 µmol·m^−2^·s^−1^, (**B**): 88.2 µmol·m^−2^·s^−1^, (**C**): 123.9 µmol·m^−2^·s^−1^, (**D**): 166.2 µmol·m^−2^·s^−1^) and fitted growth model by Van Dyck et al. [23] (Equation (5), Table 6), represented as growth curves, starting with the highest and lowest plant density variants. The simulation is conducted over a period of 80 days. Green lines indicate simulated positive plant growth at plant densities below the D_L_, red lines indicate simulated negative plant growth at plant densities above D_L_. Value pairs (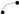) consisting of D_0_ and D_7_. Blue dashed Lines represent D_L_.

The growth data and the adapted model according to Van Dyck et al. [23] are shown in Figure 6, in analogy to Figure 5.

The growth model of Van Dyck et al. [23] has been found to possess the capacity to describe ΔD. However, the model appears to be capable of estimating growth rates with greater accuracy at plant densities that are proximate to D_L_ compared to lower densities as demonstrated in Figure 6. Notably, at low plant densities, the Van Dyck et al. [23] model tends to underestimate the growth rate. This is also reflected in the lower value of R^2^ (0.9498) in comparison to the R^2^ value of the newly developed model (0.9950), which indicates that the prediction of growth dynamics is enhanced by the newly developed model.

The newly developed model provides a reliable description of the growth dynamics over the entire range of the D-variants analyzed, which is of particular advantage given that cultivation usually takes place in the D range of roughly 10–50 gDW·m^−2^, as described below. The model can be particularly utilized in CEA cultivation, as the growth data was collected in such a cultivation system.

**Figure 7:**
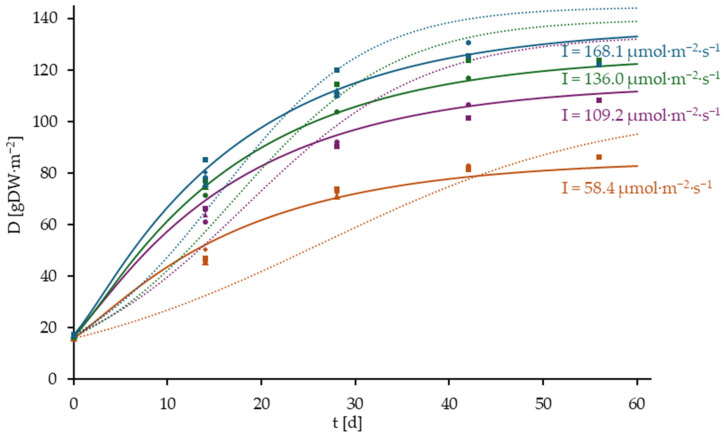
Growth data of the long-term culture trial of *L. minor* at the light intensities 58.4, 109.2, 136.0, and 168.1 µmol·m^−2^·s^−1^. The corresponding growth curves have been modeled using the developed growth model and the parameter values, except for I, are presented in Table 5 (continuous lines). The corresponding growth curves have been modeled using the growth model by Van Dyck et al. [23] and the parameter values, except for I, are presented in Table 6 (dotted lines).

The graphical representation of the growth curves of the long-term culture experiment is presented in Figure 7.

As evidenced by Figure 7, the developed growth model demonstrates a high degree of reliability in predicting the growth of a duckweed culture, as indicated by the R^2^ values of the model (58.4 µmol·m^−2^·s^−1^: 0.9816, 109.2 µmol·m^−2^·s^−1^: 0.9879, 136.0 µmol·m^−2^·s^−1^: 0.9888, 168.1 µmol·m^−2^·s^−1^: 0.9906). Furthermore, it is evident that the long-term cultures can be more accurately described by employing the developed model in comparison to the growth model proposed by Van Dyck et al. [23] (R^2^: 58.4 µmol·m^−2^·s^−1^: 0.8104, 109.2 µmol·m^−2^·s^−1^: 0.8754, 136.0 µmol·m^−2^·s^−1^: 0.9219, 168.1 µmol·m^−2^·s^−1^: 0.9281)

### 3.5. Results of the Model Utilization for Process Optimization

**Figure 8:**
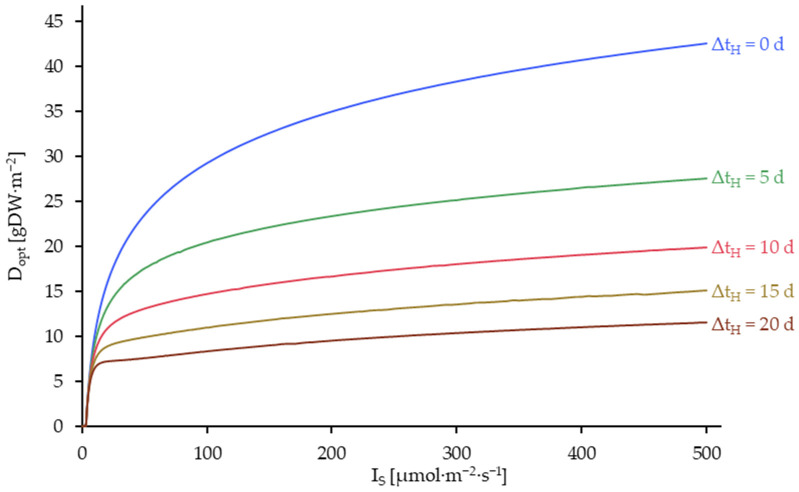
Optimum plant density after the harvesting process (D_opt_) as a function of the light intensity on the crop surface (I_S_) and the harvesting interval (Δt_H_). Simulated up to 500 µmol·m^−2^·s^−1^ and at harvest intervals (Δt_H_) of 0, 5, 10 and 20 days. Δt_H_ = 0 is equivalent to a continuous harvesting process.

The optimum plant densities for biomass production, which remains after a harvesting event in the duckweed culture (D_opt_) were calculated depending on the light intensity at different simulated harvest intervals (Δt_H_). The corresponding fitted growth curves are presented in Figure 8.

As demonstrated in Figure 8, D_opt_ approaches a saturation limit with increasing light intensity. This phenomenon can be attributed to the light saturation of the duckweed with rising light intensity. It can be posited that a light toxicity effect may be observed at considerably higher light intensities [57]. This, nevertheless, is not reflected in the modeling of the light intensity effect as a Monod type function (Equation (10)).

An extension of the harvest interval results in a reduction in D_opt_. This phenomenon is attributed to the fact that in the context of continuous harvesting (Δt_H_ = 0), it is possible to maintain the plant density at the optimum level. However, this is not feasible in the context of longer harvest intervals, resulting in the plant density being harvested below the optimum level and subsequently growing to a density above the optimum level until the next harvesting event.

**Figure 9:**
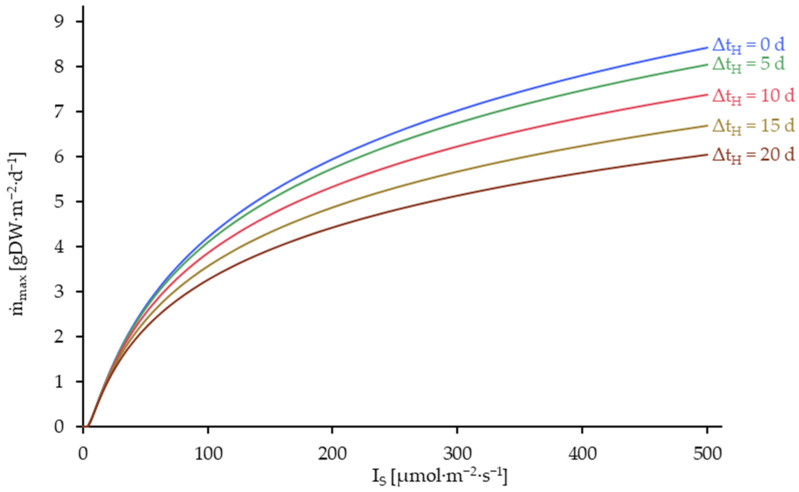
Average daily harvest yield at density-optimized harvesting process (ṁ_max_) as a function of the light intensity on the crop surface (I_S_) and the harvesting interval (Δt_H_), corresponding to the D_opt_-data given in Figure 7. Simulated up to 500 µmol·m^−2^·s^−1^ and at harvest intervals (Δt_H_) of 0, 5, 10 and 20 days. Δt_H_ = 0 is equivalent to a continuous harvesting process.

If the plant density, determined by the light intensity and the harvest interval, is defined according to Figure 8, the resulting average daily harvest yield (ṁ_max_) can be computed. The data is shown in Figure 9.

As illustrated in Figure 9, the relationship between ${\dot{m}}_{\max}$ and light intensity is subject to a saturation effect, resulting from the light saturation of duckweed. In the context of a continuous harvest (Δt_H_ = 0), it is possible to maintain the plant density at the optimal level, thereby ensuring a maximum average daily yield. However, extending the harvest interval invariably leads to a reduction in the quantity harvested.

In the context of CEA cultivation, it is imperative to consider factors beyond harvest yield, particularly with respect to the high energy input demanded by artificial lighting. The key consideration is the optimal utilization of energy. Consequently, the ratio of light energy utilized to the usable biomass is of great importance. Assuming a proportionality between the energy input and the light intensity at the cultivation surface (I_S_), the LUE can be calculated using Equation (18).

(18) $$\mathrm{LUE}=\frac{{\dot{m}}_{\max}\left({\Delta t}_{H},I_{S}\right)}{I_{S}}\;\;\;\;\;\;\;\;\;\;\;\;\;\left[\frac{\mathrm{gDW}}{\mathrm{mmol}}\right]$$

**Figure 10:**
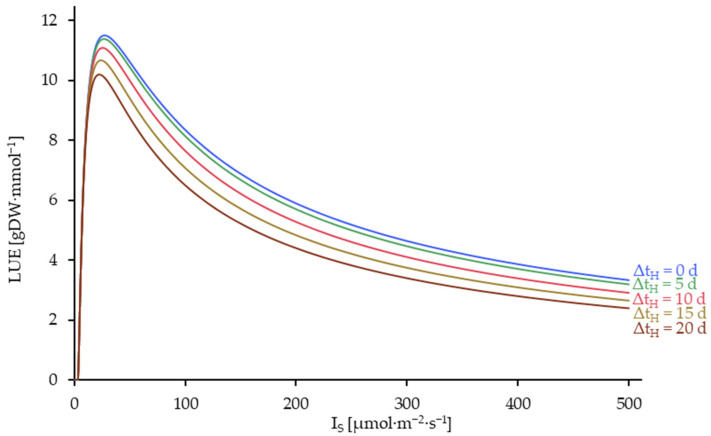
Light Use Efficiency (LUE) as a function of the light intensity on the crop surface (I_S_) and the harvesting interval (Δt_H_) in a density-optimized harvesting process, corresponding to Figure 8. Simulated up to 500 µmol·m^−2^·s^−1^ and at harvest intervals (Δt_H_) of 0, 5, 10 and 20 days. Δt_H_ = 0 is equivalent to a continuous harvesting process.

The dependence of the LUE on the harvesting interval and the light intensity LUE(Δt_H_,I_S_) is displayed in Figure 10.

The analysis of the data from Figure 10 demonstrates that the LUE is also at its maximum with continuous harvesting. However, irrespective of the harvest interval, all curves demonstrate a peak in LUE at a level of less than 27 µmol·m^−2^·s^−1^. At this point, the conversion factor of the light energy utilized to the dry mass of the duckweed is maximized.

To consider the overall efficiency of the production process, whether in terms of energy or cost efficiency, a quotient of the mass output (${\dot{m}}_{\max}$) and the energy or financial inputs should be formed. In addition to the resources utilized for lighting, these should also include additional inputs, which are made up of the energy or cost requirements of other components of the production process. These should include, for instance, the energy expended for pumps or the maintenance costs, which are not necessarily contingent on the quantity of harvest.

**Figure 11:**
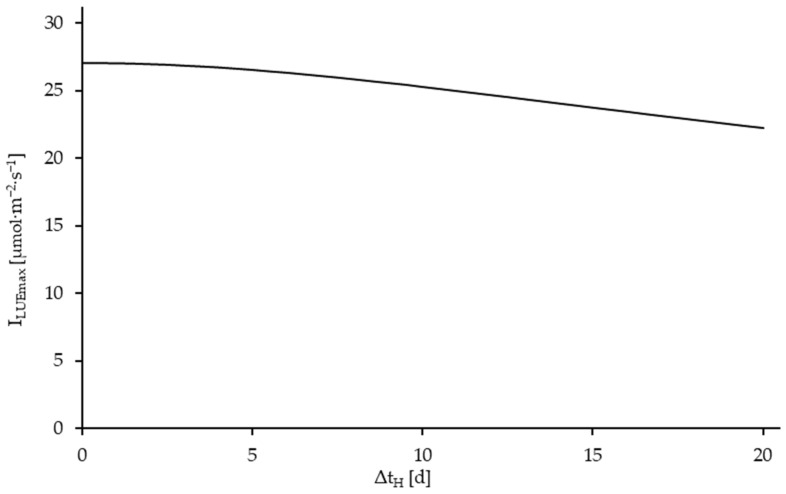
Light intensity with maximized Light Use Efficiency (I_LUEmax_) as a function of the harvesting interval (Δt_H_), corresponding to the LUE data given in Figure 10. Simulated for harvest intervals of 0–20 days.

The maximum global value of the dependency of the LUE on I_S_ is denoted by LUE_max_. The corresponding light intensity I_LUEmax_ is dependent on the harvest interval, as shown in Figure 11.

The light intensities at maximum LUE are relatively low at values below 27 µmol·m^−2^·s^−1^, which has a corresponding effect on the expected crop yields, which are comparatively low at these light intensities at less than 2 gDW·m^−2^·d^−1^ (Figure 9). Concurrently, the pure light use efficiency is highest in this instance, which illustrates the necessity to incorporate further inputs in the production process, thereby shifting the point of maximum efficiency to higher light intensities and thus higher yields.

### 3.6. Results of the Crude Protein Content

**Figure 12:**
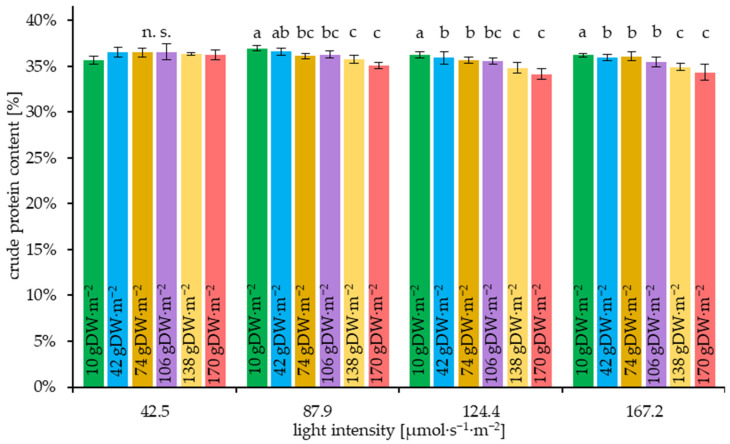
Crude protein contents (Dumas-method) of the *L. minor* experimental variants after 7 days of trial duration, grouped according to initial plant density (D_0_) and light intensity I. Mean and standard deviation. a–c: significance groups within the light intensity variants. *p* = 0.05, n. s.: not significant. Kruskal-Wallis test.

The measured crude protein contents are shown graphically in Figure 12, grouped by light intensity and initial density variants.

As demonstrated in Figure 12, a higher plant density resulted in a reduced crude protein content at medium-high and high light intensities. However, at the low light intensity variant of 42.5 µmol·m^−2^·s^−1^, no significant differences in crude protein content were observed.

The minor effect of the variation of light intensity within a comparatively low range on the crude protein content of duckweed has also been observed in other studies [16,58]. Contrarily, the comparison of low (50 µmol·m^−2^·s^−1^) and high (850 µmol·m^−2^·s^−1^) light intensities showed a significant protein content increase in *L. minor* [59].

The protein content does not significantly depend on light intensity at the same initial plant density, whereas the growth rate shows considerable variation under these conditions. This suggests that even with enhanced plant growth, an adequate nutrient supply, particularly nitrogen, was ensured, highlighting the functionality of the cultivation system used. In general, however, the differences in protein content of up to 2.9% achieved by the effects of light intensity and plant density can be classified as relatively small in contrast to the differences in growth dynamics.

The study posits that cultivating at light intensities below 30 µmol·s^−1^·m^−2^ would optimize efficiency. A substantial impact of light intensity on protein content was observed at levels above 87.9 µmol·s^−1^·m^−2^.

Consequently, the protein content is not considered in the context of maximizing efficiency during production.

In the context of agricultural production, the incorporation of additional cost and energy inputs into the overall efficiency calculation, beyond light intensity, or a decline in electricity prices, has the potential to enhance the profitability of augmenting light intensity, thereby increasing the crop yield (Figure 9). In this particular instance, it is not advisable to cultivate at a plant density that exceeds 45 gDW·m^−2^, as demonstrated in Figure 8. In this instance, as illustrated in Figure 12, the manufacturing conditions would not result in a substantial reduction in protein content. Therefore, the effect of plant density on the protein content for the purpose of efficiency-optimized production of duckweed protein is not a necessary consideration.

Furthermore, it has already been shown that other environmental factors, such as the nitrogen content or the nitrate-ammonium ratio of the nutrient solution, effectively influence the protein content of duckweed [26]. However, as these interactions were not investigated, the protein content was not directly incorporated into the growth model.

The model is primarily developed for CEA systems. Here, the fluctuations in the environmental conditions are low due to the close-meshed control. Operational complexity (fluctuating energy prices, availability of workforce, etc.) can be dealt with well using model-based planning. The model developed can provide a basis for this.

In this context, the extent to which the results can be transferred to less controllable environmental conditions, such as outdoor cultivation, must be examined. In this case, close monitoring of the environmental parameters is of great importance.

### 3.7. Limitations

In consideration of the elevated proportion of walls in relation to the cultivation area, as opposed to larger tanks that are more conducive to practical applications, it is possible that wall effects have emerged. However, these effects were not incorporated into the growth model. As outlined in Section 2, these effects were mitigated. However, their complete exclusion remains unfeasible.

The experiments were also carried out with uncontrolled air CO_2_ content and constant temperature and flow velocity. It can be assumed that these factors can also influence the growth dynamics.

It also remains to be investigated to what extent the results can be transferred to other clones and species. Since the same physiological and physical processes within the duckweed plant layer can be assumed, it can be hypothesized that the model is generally transferable. However, the values for the model parameters determined in the model fitting could be species- or clone-specific.

## 4. Conclusions

The present study offers valuable insights into the growth dynamics of *L. minor* in Controlled Environment Agriculture (CEA) systems and provides insights into the physical processes that determine the intraspecific competition for the light of the plants. Furthermore, the photosynthesis and respiration rates, along with the attenuation of light intensity in the plant biomass and the utilization of light, can be combined into a single parameter.

The results demonstrate that both plant density and light intensity exert a significant influence on the growth of *L. minor* The development of the mathematical model, which integrates these two factors, has provided a solid foundation for optimizing the cultivation process at varying light intensities and harvest intervals in a yield- and efficiency-oriented manner. The study permits well-founded assertions to be made regarding the efficiency of light use, thereby enabling the optimization of not only the harvest quantity but also the energy efficiency of such a CEA system. This could potentially result in a significant reduction of production costs and enable knowledge-based planning and economic evaluation of duckweed production in CEA systems.

Ultimately, the study demonstrates the potential to make CEA systems more efficient and, at the same time, more sustainable. The findings provide a valuable foundation for the further development of cultivation systems that can meet the challenges of the increasing global protein demand.

### Outlook

To comprehensively understand and describe the growth process of duckweed biomass, it is necessary to consider additional environmental factors. In the context of photosynthesis rate and light utilization efficiency, it is imperative to consider the temperature and CO_2_ content of the air to maximize the utilization of artificial lighting. As demonstrated in the model presented here, this can be achieved through the mathematical description of the physiological processes involved.

Moreover, integration of the model into a model-predictive control approach can ensure that yield potential is maximized even under variable framework conditions, as may occur in a practical production context like fluctuating energy prices or fluctuating sales opportunities.

## Figures and Tables

**Table 1:** Target and realize process parameters and environmental parameters of the experimental culture.

		Realized Values	
Parameter	Target Values	Mean	Standard Deviation
Water temperature	23 °C	22.9 °C	0.3 °C
pH	7.0	7.06	0.16
EC	700 µS·cm^−1^	683.4 µS·cm^−1^	14.9 µS·cm^−1^
Flow rate	400–600 L∙h^−1^	400–600 L∙h^−1^	-
Photoperiod	14 h/24 h = 0.5833	14 h/24 h = 0.5833	0
Culture trial duration	7 d	7 d	0

**Table 2:** Target and realized ion concentrations of the adapted N-Medium according to Petersen et al. [26].

		Realized Concentration [mg∙L^−1^]		
Nutrient	Target Concentration [mg∙L^−1^]	Mean	Standard Deviation	Method
NO_3_^−^-N	12.2	11.0	0.7	[27]
NH_4_^+^-N	3.5	5.5	0.8	[28]
PO_4_^3−^-P	3.1	2.6	0.3	[29]
K^+^	38.4	33.2	0.8	
SO_4_^2−^-S	39.3	49.3	3.6	
Mg^2+^	9.9	9.7	0.3	
Ca^2+^	53.5	62.0	2.2	
Na^+^	17.4	19.93	0.98	
Mn^2+^	0.072	0.413	0.148	
Zn^2+^	-	0.164	0.023	
Fe^3+^	0.154	0.079	0.054	
BO_3_^3−^-B	0.025	0.019	0.005	
Cu^2+^	-	0.016	0.004	

**Table 3:** Experiment variants and repetitions of the *L. minor* growth trial over a period of seven days.

Plant Density [gDW·m^−2^]	Light Intensity [µmol·s^−1^·m^−2^] n = 5
10	42.7
	88.2
	123.9
	166.2
42	42.7
	88.2
	123.9
	166.2
74	42.7
	88.2
	123.9
	166.2
106	42.7
	88.2
	123.9
	166.2
138	42.7
	88.2
	123.9
	166.2
170	42.7
	88.2
	123.9
	166.2

**Table 4:** Description of the variables and parameters as well as their units used for model development.

Variable/Parameter	Declaration	Unit
I	Light intensity as photon flux density in the photosynthetically active spectral range	µmol·m^−2^·s^−1^
I_S_	Light intensity as photon flux density in the photosynthetically active spectral range at surface of plant layer	µmol·m^−2^·s^−1^
D	Plant density as dry weight per area	gDW·m^−2^
D_L_	Limit density. Population-ecological capacity limit of the duckweed culture	
t	time	d
r_phot,i_	Intrinsic photosynthesis rate; theoretical photosynthesis rate at maximum light saturation and without interspecific competition	d^−1^
f_phot_	Limitation of the photosynthesis rate	-
fphot	Average Limitation of the photosynthesis rate of the entire duckweed culture	-
r_resp_	Respiration rate	d^−1^
E	Photoperiod	h·h^−1^
k	Half-saturation constant of the light intensity	µmol·m^−2^·s^−1^
$\tilde{h}$	Depth in plant cover	mm
h	Average thickness of plant cover	mm
ε	attenuation constant of the light intensity when penetrating duckweed biomass, in terms of h	mm^−1^
$\hat{\varepsilon }$	attenuation constant of the light intensity when penetrating duckweed biomass, in terms of D	gDW^−1^·m^2^
c	Proportionality constant of h and D	mm·gDW^−1^·m^2^

**Table 5:** Optimized parameter values of developed growth model expressed by Equation (17), with *p*-values and standard errors from hessian approximation.

Parameter	Initial Parameter Estimates	Value	Unit	*p*-Value	Std.-Error
r_phot,i_	0.5	0.6965	d^−1^	0.000	0.00879
r_resp_	0.05	0.0583	d^−1^	0.000	0.00152
$\hat{\varepsilon }$	0.1	0.1209	m^2^·gDW^−1^	0.000	0.00034
k	20	17.2640	µmol·m^−2^·s^−1^	0.000	0.54400
E	-	14·24^−1^ = 0.5833	h·h^−1^	-	-
R^2^	-	0.9950	-	-	-

**Table 6:** Optimized parameter values of the model by Van Dyck et al. [23] expressed by Equation (4), with *p*-values and standard errors from hessian approximation.

Parameter	Initial Parameter Estimates	Value	Unit	*p*-Value	Std.-Error
r_phot,i_	0.2	0.4602	d^−1^	0.000	0.00286
r_resp_	0.02	0.0788	d^−1^	0.000	0.0030
h_D_	70	223.7906	m^2^·gDW^−1^	0.000	3.7984
K_I_	30	50.4300	µmol·m^−2^·s^−1^	0.000	3.1935
E	-	14·24^−1^ = 0.5833	h·h^−1^	-	-
R^2^	-	0.9498	-	-	-

## Data Availability

The original contributions presented in this study are included in the article/Supplementary Material. Further inquiries can be directed to the corresponding author.

## Supplementary Materials

The following supporting information can be downloaded at: https://www.mdpi.com/article/10.3390/plants14111722/s1, Figure S1: Light Distribution Basin 2, Figure S2: Light Distribution Basin 3, Figure S3: Light Distribution Basin 4, Figure S4: Light Distribution Basin 5, Figure S5: Light Distribution Basin 6, Figure S6: Light Distribution Basin 7, Figure S7: Light Distribution Basin 8, Figure S8: Light Distribution Basin 9, Table S1: Light Distribution Basin 2, Table S2: Light Distribution Basin 3, Table S3: Light Distribution Basin 4, Table S4: Light Distribution Basin 5, Table S5: Light Distribution Basin 6, Table S6: Light Distribution Basin 7, Table S7: Light Distribution Basin 8, Table S8: Light Distribution Basin 9, R-Code S1: Mean Light Intensity Calculation Basin 2, R-Code S2: Mean Light Intensity Calculation Basin 3, R-Code S3: Mean Light Intensity Calculation Basin 4, R-Code S4: Mean Light Intensity Calculation Basin 5, R-Code S5: Mean Light Intensity Calculation Basin 6, R-Code S6: Mean Light Intensity Calculation Basin 7, R-Code S7: Mean Light Intensity Calculation Basin 8, R-Code S8: Mean Light Intensity Calculation Basin 9, R-Code S9: Statistics Main Experiment New Model, R-Code S10: Statistics Main Experiment Van Dyck Model, R-Code S11: Statistics Validation Experiment New Model, R-Code S12: Statistics Validation Experiment Van Dyck Model.
