# Supplementary figures and images for: Modeling Growth Dynamics of Lemna minor: Process Optimization Considering the Influence of Plant Density and Light Intensity
Source: Plants (Basel). 2025 Jun 5;14(11):1722. doi: 10.3390/plants14111722 (PMC12157068; doi:10.3390/plants14111722)

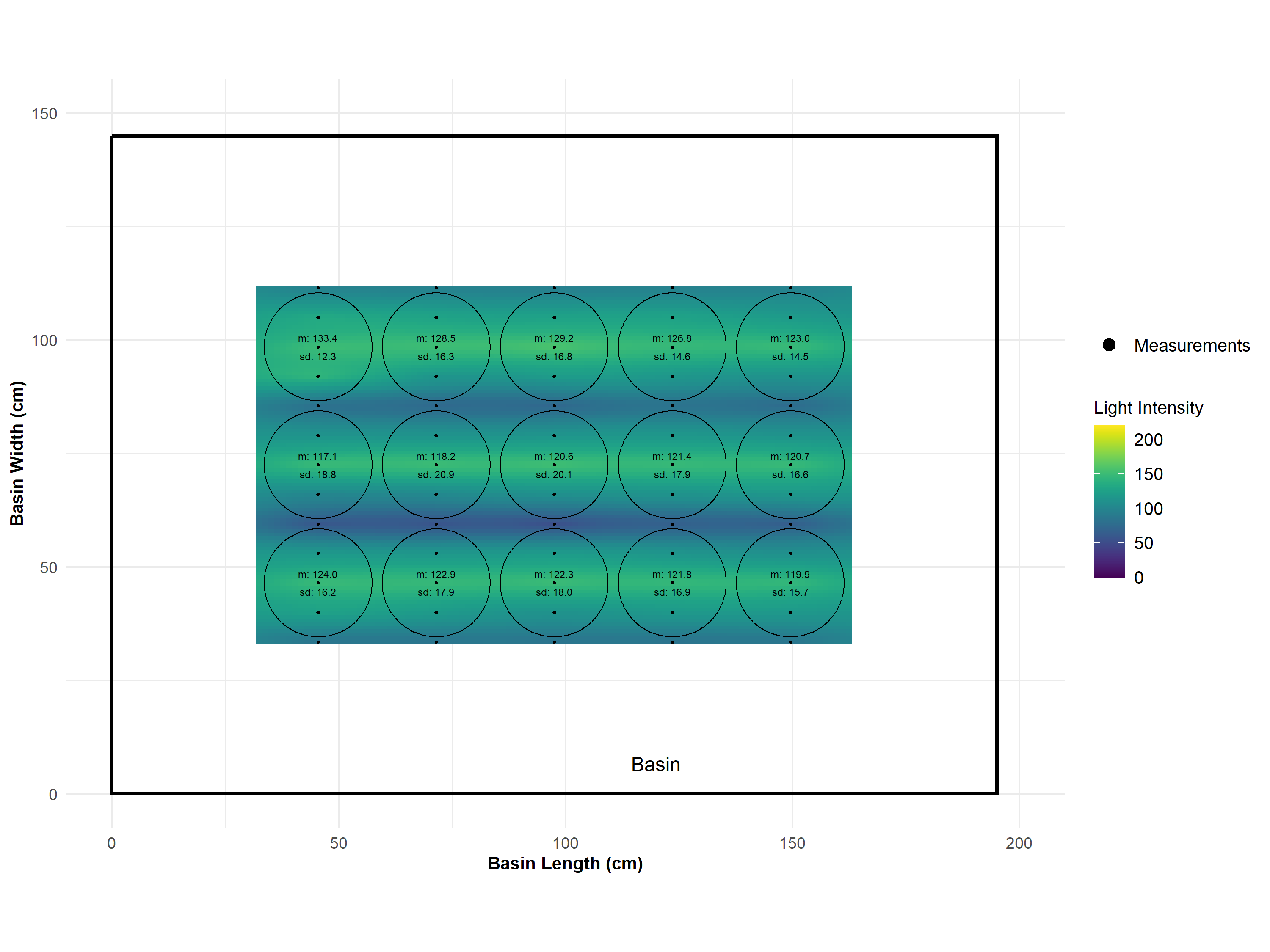

Supplement: Supplementary file 1 [file plants-14-01722-s001.zip › Figure S1 Light Distribution Basin 2.png]

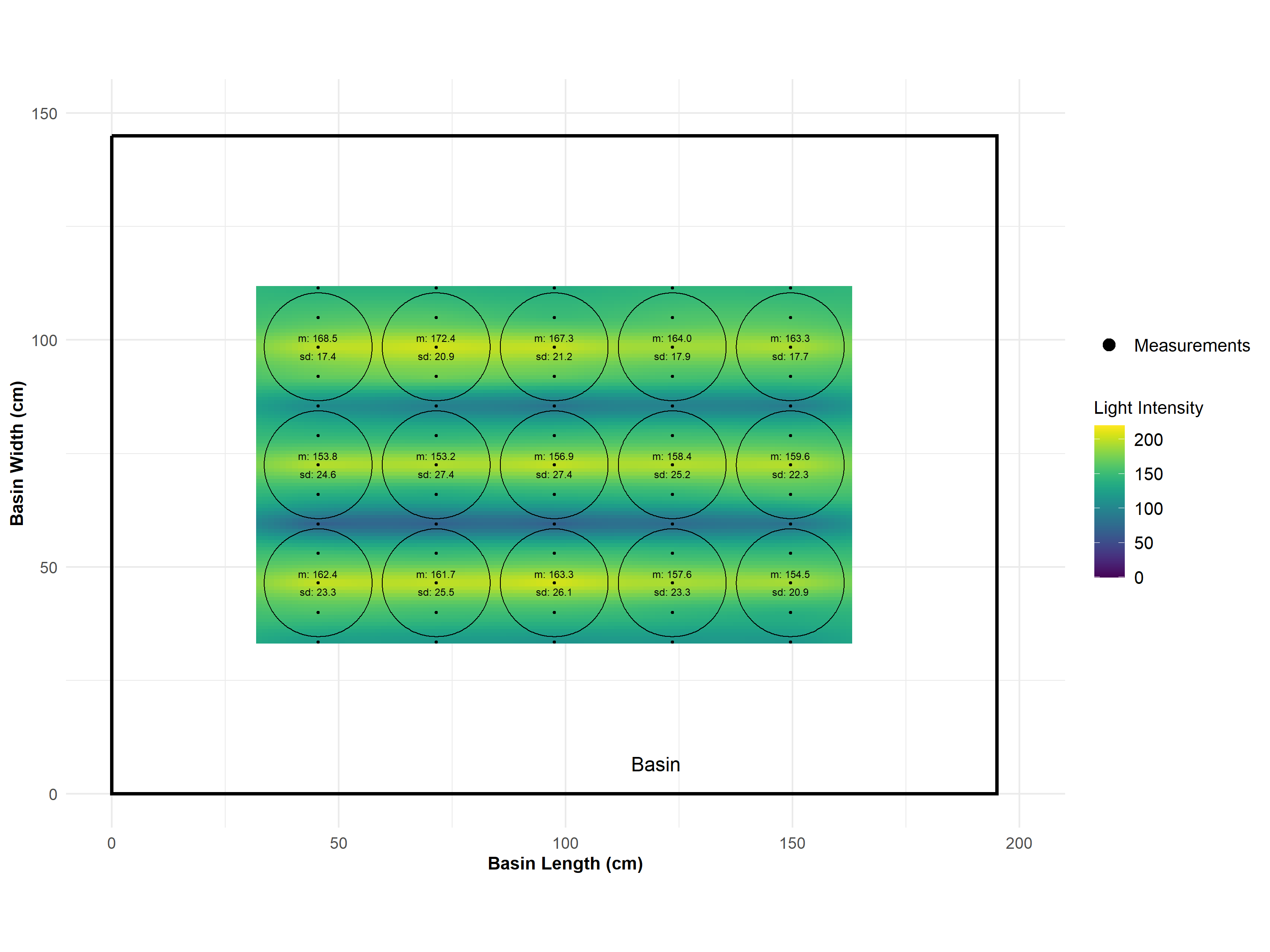

Supplement: Supplementary file 1 [file plants-14-01722-s001.zip › Figure S2 Light Distribution Basin 3.png]

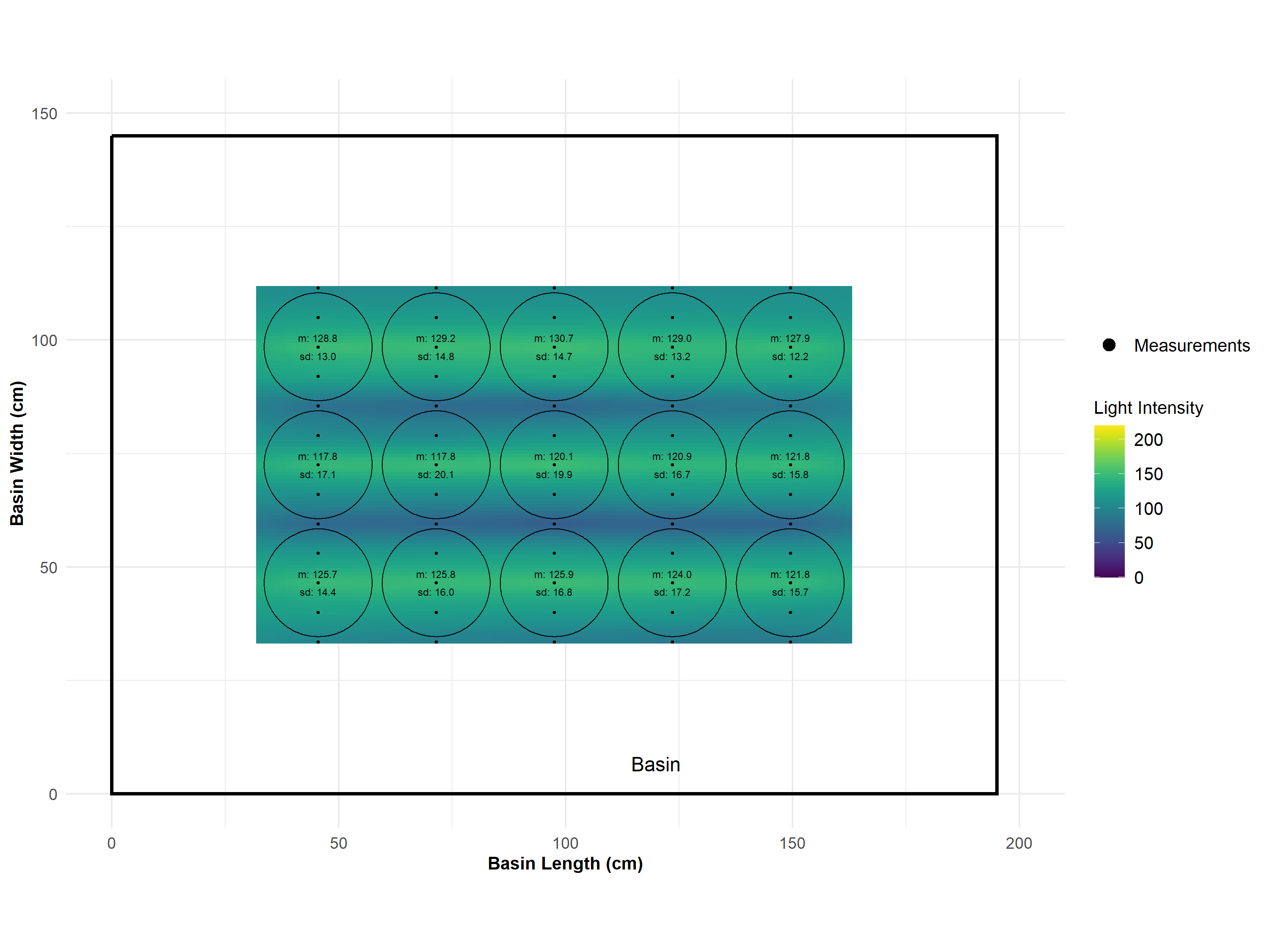

Supplement: Supplementary file 1 [file plants-14-01722-s001.zip › Figure S3 Light Distribution Basin 4.png]

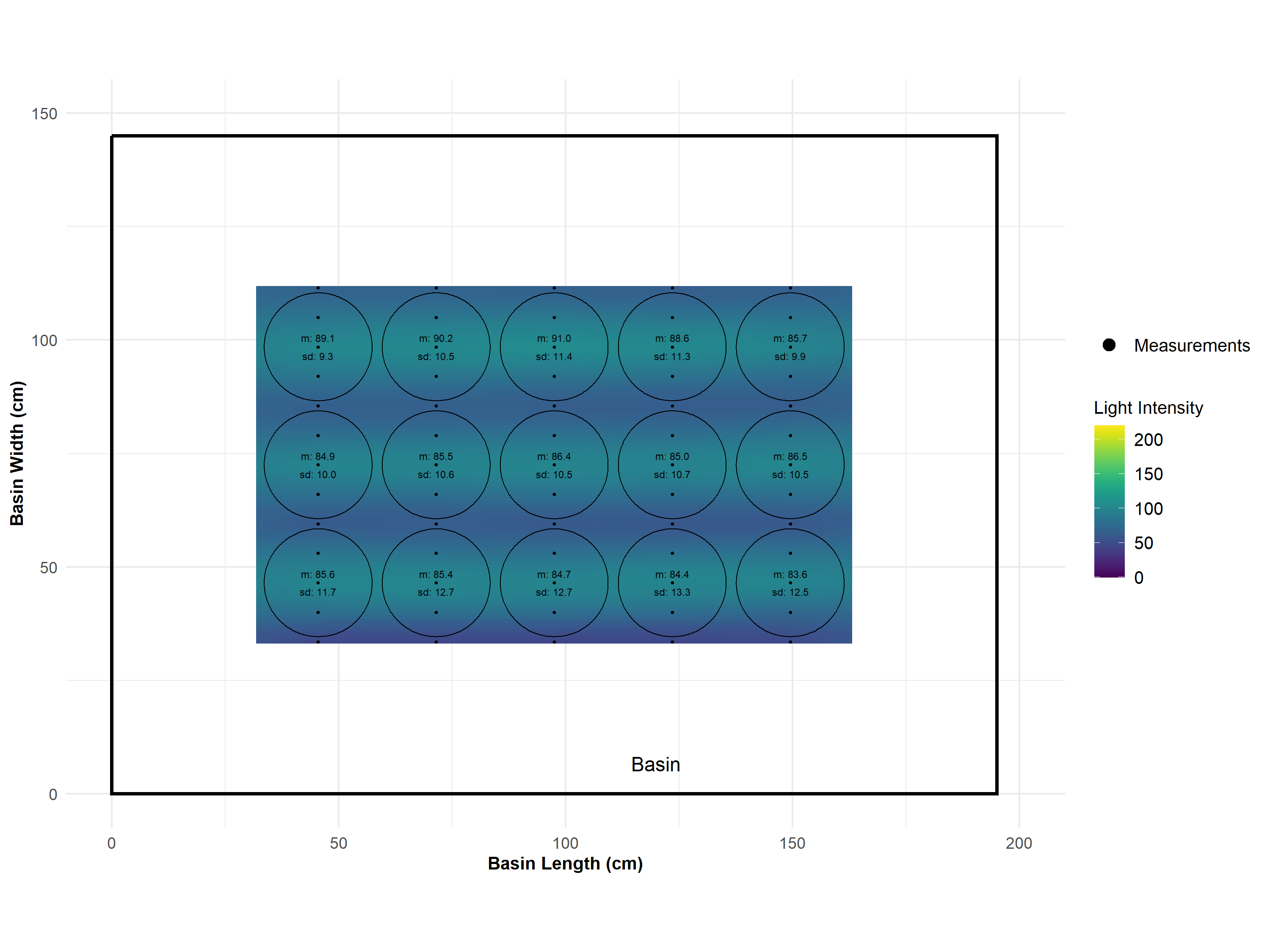

Supplement: Supplementary file 1 [file plants-14-01722-s001.zip › Figure S4 Light Distribution Basin 5.png]

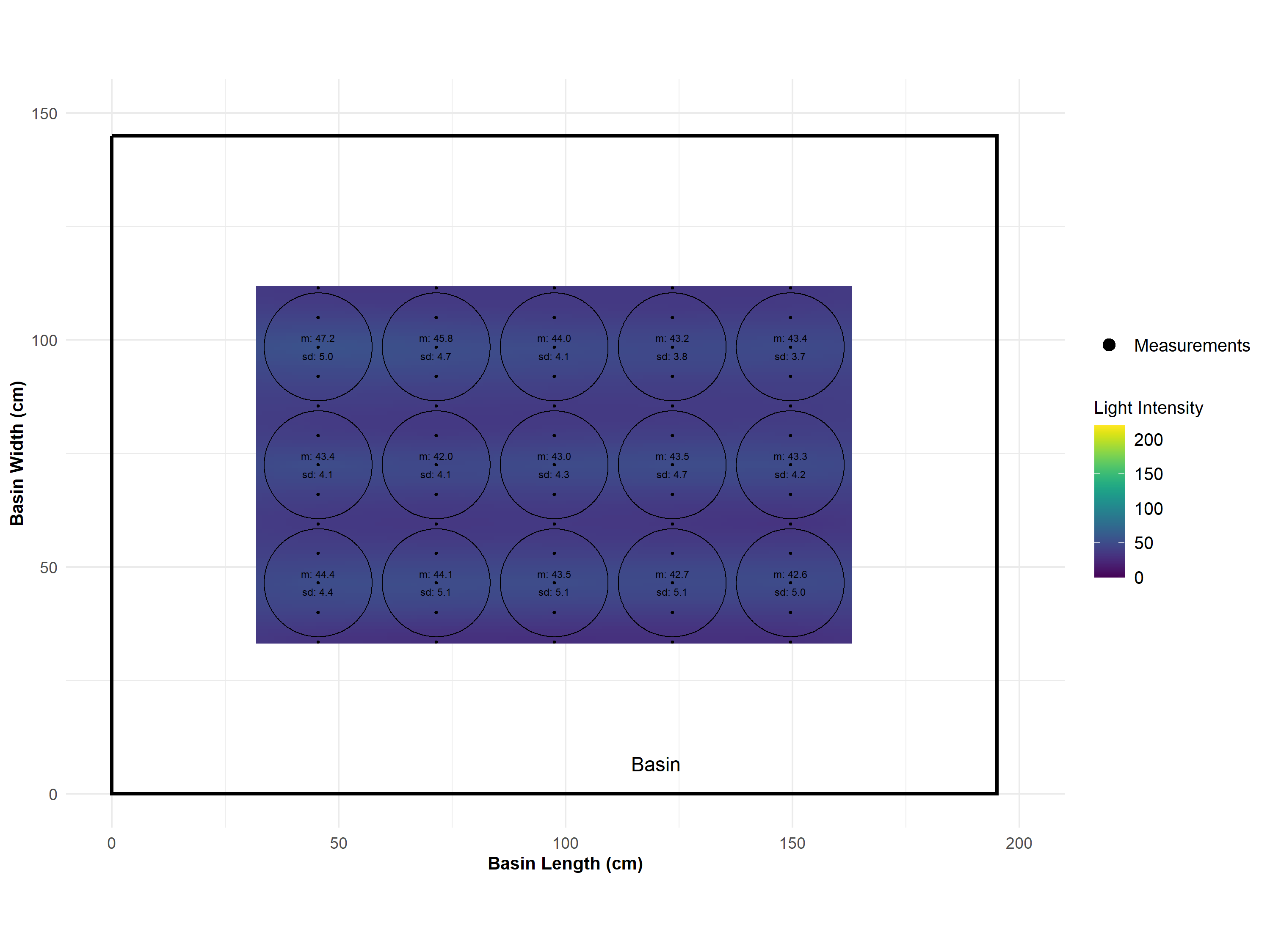

Supplement: Supplementary file 1 [file plants-14-01722-s001.zip › Figure S5 Light Distribution Basin 6.png]

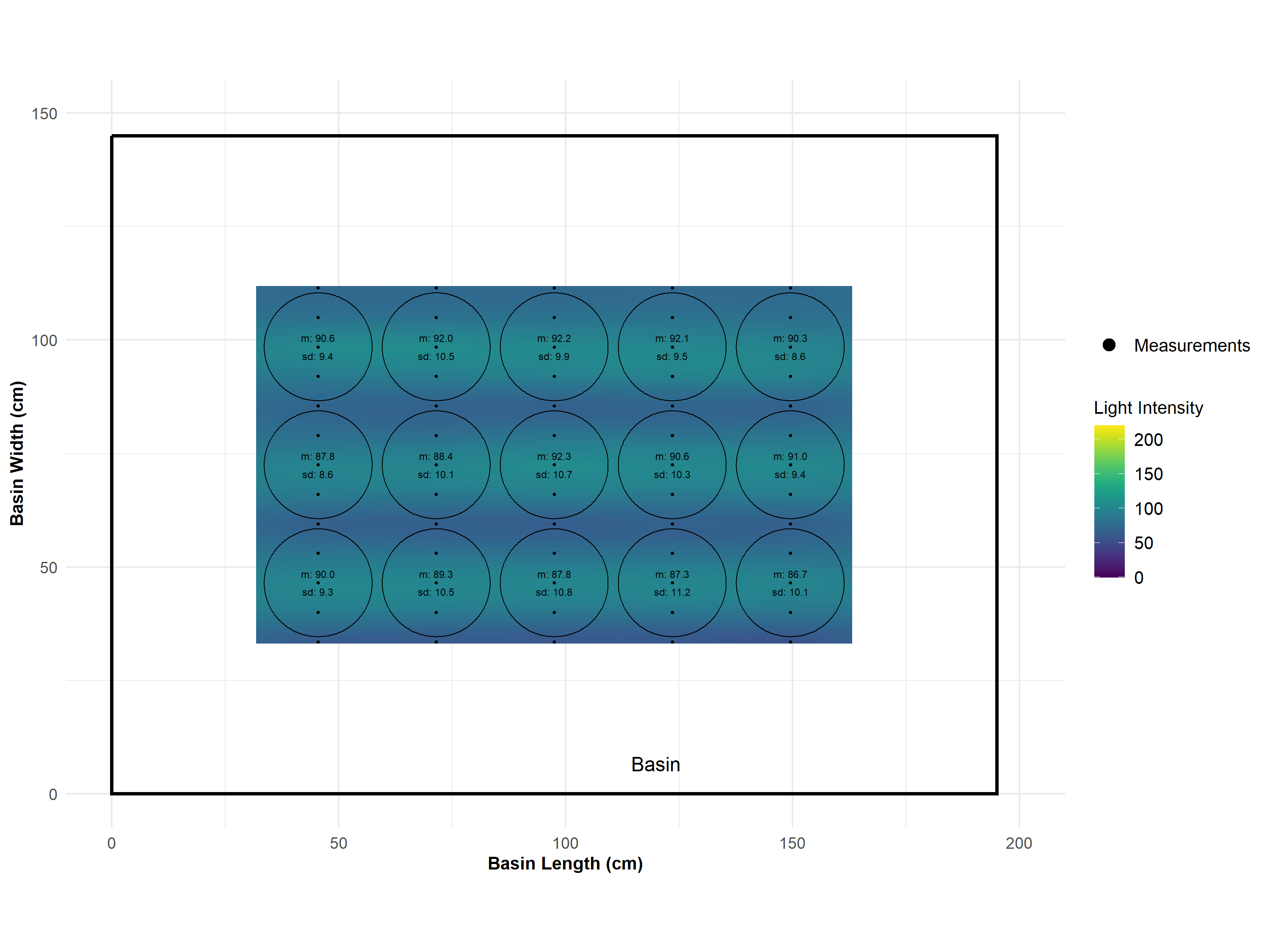

Supplement: Supplementary file 1 [file plants-14-01722-s001.zip › Figure S6 Light Distribution Basin 7.png]

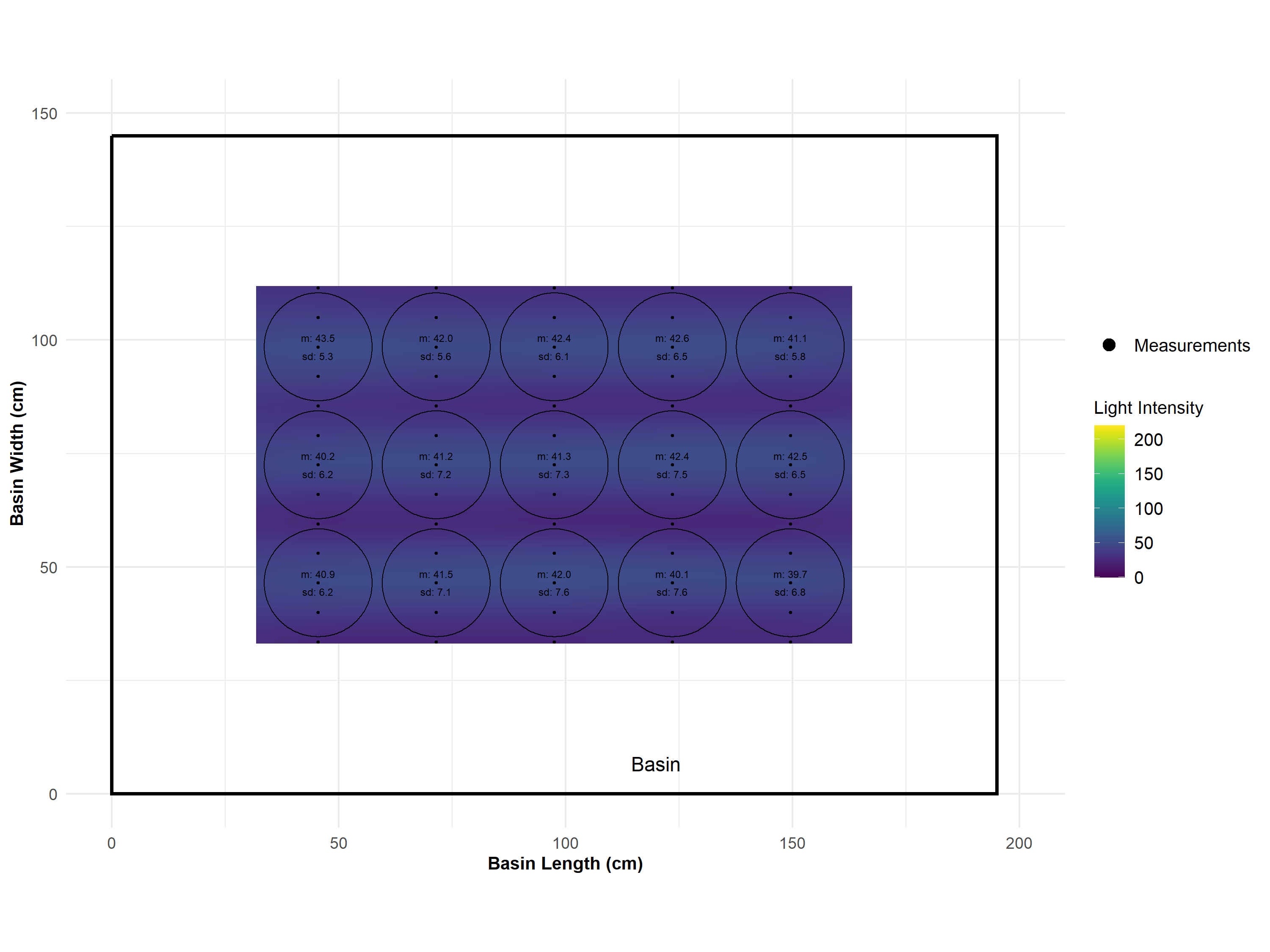

Supplement: Supplementary file 1 [file plants-14-01722-s001.zip › Figure S7 Light Distribution Basin 8.png]

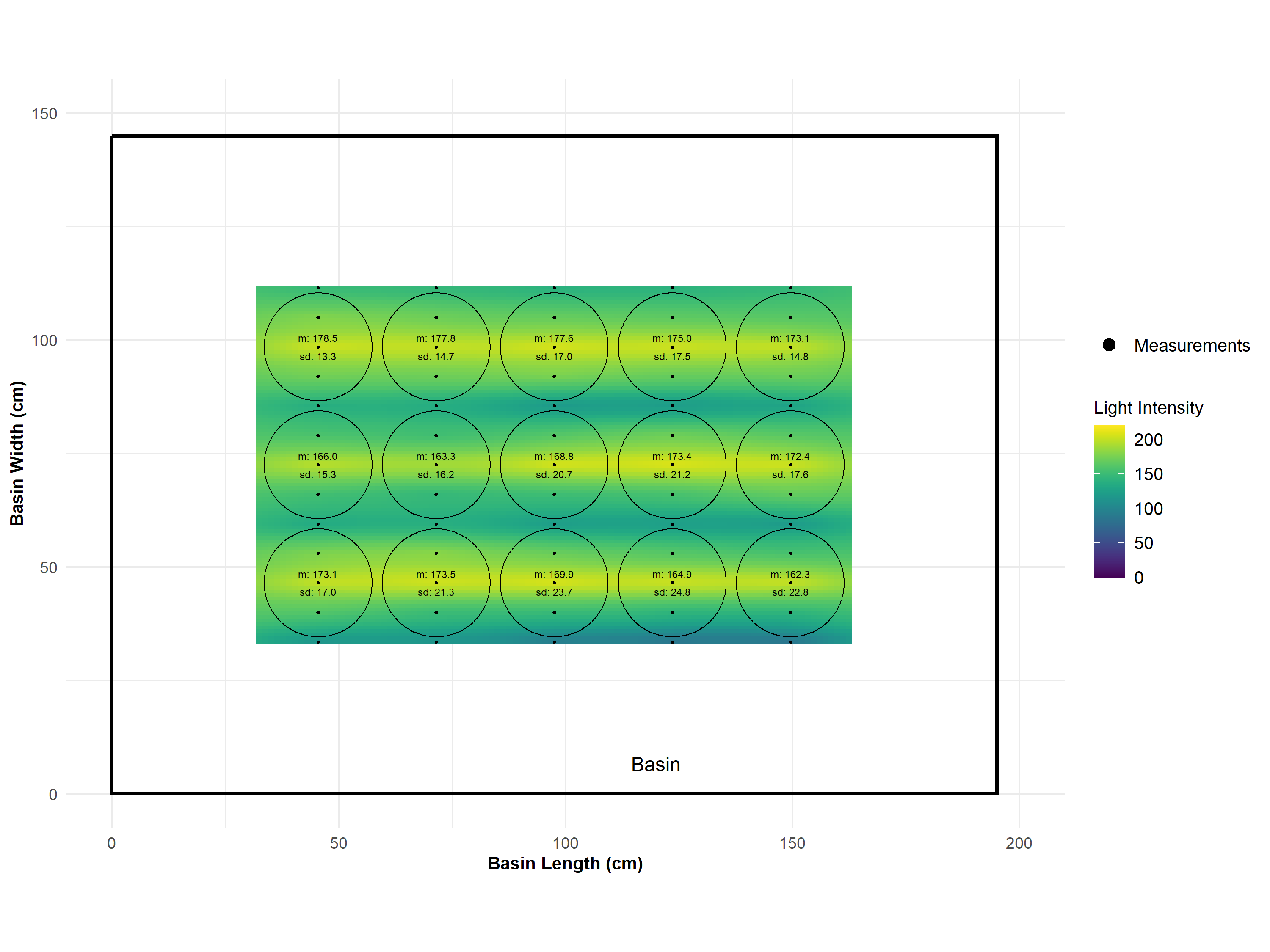

Supplement: Supplementary file 1 [file plants-14-01722-s001.zip › Figure S8 Light Distribution Basin 9.png]
